# A scoping review of the use of behavioral theories in health professionals’ continuing professional development research

**DOI:** 10.1186/s12909-025-08276-3

**Published:** 2025-12-29

**Authors:** Ola Adlan, Derek Stewart, Youmna Ahmed, Muna Al-Ismail, Heba Al-Omary, Zachariah Nazar

**Affiliations:** College of Pharmacy, QU Health, Qatar University, Doha, Qatar

**Keywords:** Behavioral theories, Continuing Professional Development (CPD), Health professionals, Self-Determination Theory (SDT), Theoretical Domains Framework (TDF), Theory of Planned Behavior (TPB)

## Abstract

**Introduction:**

Continuing Professional Development (CPD) is critical for maintaining healthcare professionals' competencies and ensuring high-quality care. Despite its importance, the application of behavioral theories in CPD is limited. This scoping review maps the use of behavioral theories in CPD for health professionals, identifies gaps, and provides recommendations for future research.

**Method:**

Joanna Briggs Institute guidelines and the PRISMA-ScR framework guided this review. Databases including PubMed, Embase, CINAHL, ERIC, APA PscyARTICLES and ProQuest Central were searched up to September 2024. Two independent reviewers screened and assessed the articles, with disagreements resolved through consensus. A total of 4,051 records were retrieved, 1,900 were screened after duplicates were removed, and 12 studies reporting the use of behavioral theory in CPD met the inclusion criteria.

**Results:**

Of the 12 included studies, the Theory of Planned Behavior was the most frequently applied theory. Behavioral theories were often used superficially for questionnaire design or predicting CPD participation. There was limited use of comprehensive frameworks like the Theoretical Domains Framework and minimal representation of healthcare professions other than pharmacists.

**Discussion:**

There is limited use of behavioral theories in CPD research and a lack of diverse study designs. Most studies applied behavioral theories superficially, without fully leveraging their potential to guide intervention development and evaluation. The findings align with previous research, suggesting a stronger theoretical foundation is needed to enhance understanding of CPD outcomes.

**Conclusion:**

There is a need for more rigorous research designs, greater theoretical justification, and broader inclusion of healthcare professions in CPD research. Future studies should adopt multi-theoretical frameworks and employ longitudinal and randomized controlled trial designs to better understand the impact of behavioral theories on CPD outcomes. Such advancements will help develop more effective CPD interventions, ultimately improving health professionals' performance and patient care.

**Supplementary Information:**

The online version contains supplementary material available at 10.1186/s12909-025-08276-3.

## Introduction

Continuing professional development (CPD) is a critical aspect of health professionals' lifelong learning, ensuring they remain competent, knowledgeable, and capable of providing high-quality care in rapidly evolving health environments [1].

While Continuing Education (CE) typically refers to structured learning activities focused on maintaining or updating professional knowledge, CPD represents a broader, self-directed and reflective process encompassing lifelong learning and continuous improvement in professional competence [2, 3]. CPD includes a wide range of educational activities, such as formal education programs, workshops, conferences, and self-directed learning. These activities aim to maintain and enhance the knowledge, skills, and competencies necessary for professional practice [4], and to foster continuous improvement in patient outcomes and health services in a culture of lifelong learning [5].

However, despite its recognized importance, the effectiveness of CPD in achieving these goals is inconsistent. Evidence suggests significant variability in the impact of CPD on health professionals' behavior and, consequently, on patient care outcomes [1, 4, 6]. A persistent challenge in CPD is the translation of newly acquired knowledge and skills into clinical practice, which is influenced by various factors, including individual motivation, workplace culture, and personal beliefs and attitudes [7–10].

Behavioral theories provide valuable frameworks for understanding the factors that impact the successful implementation of CPD into clinical practice. These theories, widely applied in fields such as psychology, education, and public health, offer insights into the determinants of behavior change and support the design of interventions that enhance CPD effectiveness [11, 12]. A 2024 scoping review investigating the reported behavioral theories/models/frameworks used in pharmacy practice research revealed the Theory of Planned Behavior (TPB) to be the most frequently adopted [13]. The TPB posits that attitudes, subjective norms, and perceived behavioral control influence an individual's intentions and subsequent actions [14, 15]. Applying such theories to CPD helps educators identify key factors that affect health professionals' engagement with CPD activities and their application of new knowledge and skills in clinical settings [16].

Self-Determination Theory (SDT) is another behavioral theory that has been utilized within health education research. SDT emphasizes the role of intrinsic and extrinsic motivation in driving behavior, suggesting that behaviors driven by intrinsic motivation are more likely to result in sustained behavior change than those driven by external pressures or rewards [17, 18]. In the context of CPD, understanding motivational factors that encourage health professionals to engage in lifelong learning is crucial [19]. Evidence from SDT-informed CPD interventions suggests potential for improved engagement and sustained learning outcomes, although causal effects remain unclear [20].

Despite the recognized benefits of incorporating behavioral theories into CPD, there is limited research systematically exploring how these theories are used to enhance CPD activities for health professionals. Published reviews within the CPD literature suggest that while some studies incorporate behavioral theories into CPD, this approach is not widespread, and considerable variation exists in how these theories are applied [21–23]. Notably, many studies that use behavioral theories often focus on specific aspects of CPD, such as motivation or attitude change, rather than adopting a comprehensive approach that integrates multiple behavioral constructs to address the complexities of professional learning and behavior change [24, 25].

This scoping review aimed to address this gap by systematically mapping the use of behavioral theories in CPD for health professionals. The objectives of this review were to:

1. Describe the health professionals and settings in the included studies.
2. Characterize the behavioral theories used and the stated rationale for their selection.
3. Describe how these theories were applied in CPD activities.
4. Align the findings with the domains and constructs of the behavioral theories used.

By achieving these objectives, this review seeks to provide a comprehensive overview of how behavioral theories are currently being utilized in CPD, identify gaps in the existing literature, and offer recommendations for future research and practice.

## Methods

This scoping review was guided by the Joanna Briggs Institute (JBI) Reviewers’ Manual [26], which provides a structured approach to conducting scoping reviews, ensuring comprehensiveness and rigor in identifying and synthesizing the relevant literature. It is reported following the Preferred Reporting Items for Systematic Reviews and Meta-Analyses extension for Scoping Reviews (PRISMA-ScR) guidelines [27]. This review was not preregistered.

### Inclusion and exclusion criteria

#### Inclusion criteria

The review focused on primary research studies, including quantitative, qualitative, and mixed-methods designs, that used behavioral theory in the context of CPD for health professionals. Studies were included if they met the following criteria:

- *Participants:* Health professionals, including but not limited to physicians, nurses, pharmacists, dentists, mental health professionals, and allied health personnel.
- *Concept:* The application of behavioral theories in any stage of the CPD cycle, which includes planning, delivery, implementation, and evaluation of CPD activities.
- *Context:* Studies reporting the use of behavioral theories to understand, predict, or influence professional behaviors in CPD settings.

#### Exclusion criteria

Studies were excluded if they were not published in English, and if the study was relating to an undergraduate/post-graduate academic program such as a diploma or post-graduate certificate. Additionally, book chapters, reviews, conference abstracts, and articles with no available full text and non-peer-reviewed materials were excluded. Grey literature was explored via ProQuest and Google Scholar, but non-peer-reviewed materials were excluded from the final synthesis to ensure methodological rigor.

### Search strategy

A comprehensive search strategy was developed in collaboration with a medical librarian. The search was designed to capture all relevant studies from the inception of the databases to May 2023 and updated in August 2024. The databases searched included PubMed, Embase, the Cumulative Index to Nursing and Allied Health Literature (CINAHL), and the Education Resources Information Center (ERIC) which were accessed via EBSCO; and American Psychological Association (APA) PsycARTICLES. Grey literature was also searched using ProQuest Central and Google Scholar to identify any additional studies that may not have been captured in the primary database search. Minor variations in retrieved article counts may occur due to ongoing database indexing updates since the initial search.

The search terms were organized into three main categories: (1) health professionals, (2) CPD, and (3) behavioral theories. Boolean operators (AND, OR) were used in combination with truncation and phrase searching to enhance the retrieval of relevant studies (see Appendix 1 for full details of the search).

All search results were exported to EndNote X8 for reference management, and duplicates removed prior to screening. Rayyan was used for independent title/abstract and full-text screening, and data extraction was conducted using standardized Excel templates.

### Study selection

**Fig. 1:**
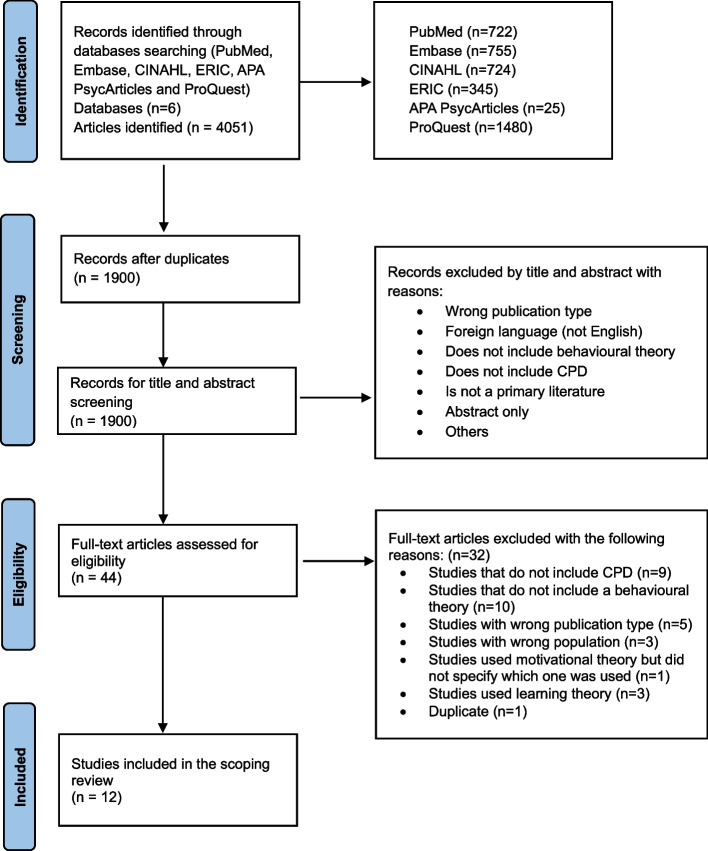
PRISMA flow chart of scoping review process

The study selection process involved two stages: (1) title and abstract screening and (2) full-text screening. All titles and abstracts identified from the search were screened independently by two reviewers using the inclusion and exclusion criteria. Discrepancies between the reviewers were resolved through discussion, and a third reviewer was consulted if consensus could not be reached. Articles that met the inclusion criteria were retrieved for full-text screening by the same two reviewers working independently, who later met to reach consensus on discrepancies. For articles where consensus was not reached between the two reviewers, opinion was taken from a third reviewer. The reasons for exclusion at the full-text screening stage were documented and presented in a PRISMA flow diagram (Fig. 1).

### Data extraction and synthesis

Data were extracted using a standardized data extraction form developed by the research team. The form captured key study characteristics, including author(s), year of publication, country, study design, health professional(s) involved, behavioral theory used, rationale for theory selection, and how the theory was applied in the study. The extraction process was carried out independently by two reviewers, with any disagreements resolved through discussion or by consulting a third reviewer.

The descriptive synthesis aimed to provide insights into how behavioral theories have been used to guide or inform CPD interventions. This involved counting the frequency of specific behavioral theories used across the included studies, and examining the methods of application, and the impact of these theories on the outcomes of CPD activities. The results were organized to align with the domains and constructs of the behavioral theories identified in the included studies. Domains were predefined based on the core constructs described in each behavioral theory. During data extraction, reviewers mapped study variables to these predefined domains to maintain consistency and ensure theoretical alignment.

## Results

### Search results

The database search yielded a total of 4,051 records. After the removal of duplicates, 1,900 unique articles remained. No further studies were identified through the grey literature search. These articles were subjected to title and abstract screening, which resulted in the exclusion of 1,856 articles based on the predefined inclusion and exclusion criteria. The full texts of the remaining 44 articles were then assessed for eligibility, leading to the exclusion of 32 articles for reasons such as irrelevance to CPD, lack of application of behavioral theories, or wrong study design or population. Ultimately, 12 studies were included in this scoping review. The study selection process is detailed in the PRISMA flow diagram (Fig. 1).

### Descriptive summary of included studies

The 12 studies included in this review were published between 2007 and 2023, with the largest number of studies being conducted in the Netherlands (3 studies), Canada (3 studies) and the United States (3 studies) [28*, 29*, 30*, 31*, 32*, 33*–34*, 35*]. Pharmacists were the most frequent participants in the included studies, either as a single professional group (4 studies) or as part of a multi-professional study. The sample sizes varied widely across studies, ranging from 11 to 8,997 participants, reflecting the diversity in study designs and settings (Table 1 provide a summary of the study characteristics).

**Table 1:** Summary of study characteristics

Characteristic	Number of studies
Single health profession	6
Multiple health professions	6
**Type of health professional**	
- Pharmacists	4
- Health professionals	3
- Dentists	1
- Pediatric health providers	1
- Mental health practitioners	1
- Clinicians/physicians	2
**Type of setting**	
- Primary care (clinical setting)	7
- Secondary care (academic setting)	3
- Not identified	2
**Country**	
- Netherlands	3
- United States	3
- Iran	1
- Saudi Arabia	1
- Canada	3
- United Kingdom	1

### Trend of utilization of behavioral theories in CPD

**Fig. 2:**
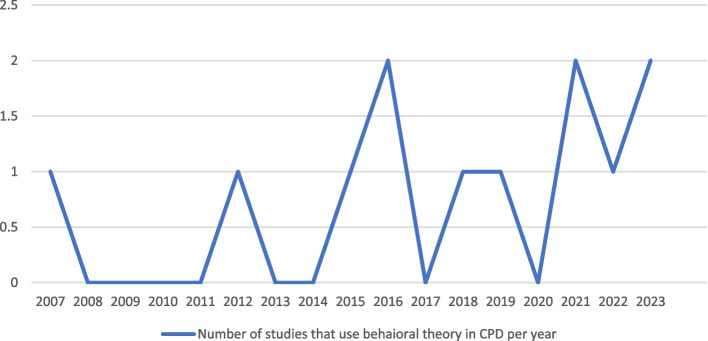
The number of studies adopting behavioral theory in CPD since 2007

From the included studies, the application of behavioral theories in CPD is seen to be increasing from over time (Fig. 2), illustrating a growing trend to utilize structured approaches for identifying motivational factors, barriers, and facilitators that might have an impact on CPD.

### Use of behavioral theories in CPD

Table 2 presents the behavioral theories reported in the included studies and the corresponding domains that the research aimed to investigate (e.g., attitudes, motivation, perceived behavioral control). For example, if a study using the Theory of Planned Behavior (TPB) examined attitudes, subjective norms, and perceived control, these were noted as the assessed domains.

**Table 2:** Behavioral theories and the assessed domain(s)

Theory	No. of Articles	Rationale (Domains Assessed)
Theory of Planned Behavior (TPB)	4	Used to explain health professionals' behaviors and attitudes. (Attitude, subjective norms, perceived behavioral control)
Self-Determination Theory (SDT)	3	Applied to study motivation in medical education settings. (Motivation)
Theoretical Domains Framework (TDF)	1	Used to assess the impact of educational workshops on behavior. (Knowledge, skills, social influences, beliefs, etc.)
Diffusion of Innovation (DoI) theory	2	Examined the spread of ideas and the influence on behavioral change. (Knowledge, attitudes, behavioral intention)
Combination of TPB, TDF, & COM-B	1	Analyzed the combined impact on behavior adoption in CPD. (Knowledge, skills, environmental context, professional role)
Combination of Godin’s Integrated Behavior Change Framework and TDF	1	Determine the factors influencing intention and behavior (Beliefs about capabilities, social influences, moral norm and beliefs about consequences)

The most frequently used behavioral theory was the Theory of Planned Behavior (TPB), which was used in 4 studies. Self-Determination Theory (SDT) was the second most commonly used, applied in 3 studies. Others included a theoretical framework, the Theoretical Domains Framework (TDF), and Diffusion of Innovation (DoI) theory. Moreover, two studies combined multiple theories; one study utilized a combination of TPB, TDF, and the Capability, Opportunity, Motivation, and Behavior (COM-B) model to examine the factors influencing behavior adoption; and the second study used a combination of Godin’s Integrated Behavior Change Framework and TDF to evaluate the factors influencing behavior change, identify barriers and facilitators, and offer insights for designing more effective CPD interventions.

### Impact and benefits of behavioral theories

A study applying SDT indicated that health professionals’ motivation to participate in CPD activities decreases over time, and high motivation does not always lead to increased participation in CPD activities [34*]. A second study using SDT identified motivational profiles and found that pharmacists whose profiles were characterised by high autonomous motivation and low controlled motivation were more likely to exhibit better educational engagement [33*, 36*].

A study employing TPB measured dentists’ attitudes, perceived social norms, perceived behavioural control and received continuing education, found that intention to manage drug users was significantly associated with perceived norms and perceived control, but not with participation in continuing education [37*].

Casper [29*] used TPB to compare a standard continuing education class versus one designed according to TPB constructs (attitudes, norms, perceived behavioural control) and found that the TPB-informed class significantly increased intentions to use a new tool (74% vs. 42%) and subsequent implementation among mental-health professionals. Walling et al. [35*], also used theory to design CPD activities,applying Diffusion of Innovations theory to test two CME modules among pediatric healthcare providers and found knowledge and intention gains, with strong links between intention and subsequent reported behaviour change in practice.

Examples of studies using multiple theories included the following:

- Rahimi et al. [38*] applied the TDF and Godin’s integrated behaviour change model (an extension of the TPB) to design and evaluate a continuing professional education workshop on shared decision-making for Iranian healthcare professionals. Using these theories to identify barriers and measure intention, the study found a significant increase in participants’ intention to engage in shared decision-making after training.
- Kanyinga et al. [31*] also combined Godin’s integrated model for health professional behaviour change and the TDF to assess how CPD courses influence physicians’ behavioural intentions and practice over time. Guided by these models, the study found that intention increased significantly after the course and that higher post-course intention predicted self-reported behaviour change at six-month follow-up.
- Tardif et al. [32*] using the TDF, COM-B and the CPD-REACTION instrument to evaluate a CPD course integrating sex and gender considerations into diabetes and depression care. Grounding the intervention in behavioural theory, the study found that participants exposed to the sex/gender-integrated module reported stronger intention and confidence to apply these concepts in clinical practice compared with controls.

Table 3 presents a summary of the theories used and the associated subsequent findings in the included studies.

**Table 3:** How theories were used and their benefits

Author, year, Country	Theory	Study design	How theory was used	Main findings in relation to theory	Benefits of using theory
[34*], Netherlands	SDT	Prospective longitudinal study	A questionnaire was adapted to measure changes in motivation for CE	Motivation for CE decreases by time because traditional CE does not seem to meet professional development needs adequately	Assessed motivation and identified factors influencing participation
[33*, 36*], Netherlands	SDT	Quantitative cross-sectional study	A questionnaire was adapted to measuring the contextual motivation for CE	High motivation does not necessarily translate into higher participation in CE activities, suggesting that the current CE system may not adequately fulfill the psychological needs for autonomy and competence	Clarify how motivation can act as facilitator or barrier to engagement in CE activities
[33*, 36*], Netherlands	SDT	Quantitative cross-sectional study	A questionnaire was used to measure the quantity and quality of motivation for CE	Four motivational profiles were identified, demonstrating that higher quality motivation (autonomous motivation) is associated with better educational outcomes, thereby supporting SDT's emphasis on the significance of intrinsic motivation over extrinsic factors in CE	The application of SDT provided a theoretical foundation for understanding pharmacists' motivations and how it can be leveraged to enhance their CE experiences
[37*], Saudi Arabia	TPB	Quantitative cross-sectional study	TPB was used to develop questionnaire assessing (attitudes, perceived norms, and perceived control)	Perceived social norms and perceived control significantly influenced intentions to adopt behavior, while attitudes had a limited role, and CE did not show a significant impact	Understanding the factors influencing intentions to adopt behavior, guided the development of targeted training interventions, identified key determinants for improving care
[39*] UK	TPB	Semi-structured interviews	TPB was used to design interview questions (attitudes towards CPD, attitudes of others towards CPD and perceived behavioral control)	Perceived barriers included lack of time to undertake CPD, not knowing what to identify for learning and not knowing how to address the learning need. The perceived benefits of undertaking CPD were clearly expressed. The complex nature of form filling was highlighted as a perceived barrier when questioned about views of pharmacy colleagues (subjective norms)	Describe attitudes and beliefs towards conducting and recording CPD as well as identifying facilitators and barriers which may help to inform the design of useful support mechanisms to conduct and record CPD effectively
[29*] US	TPB	Randomized controlled trial [Experimental Quantitative Study]	TPB was used to design a CE class	Participants in the theory-guided CE class demonstrated significantly stronger intentions to implement and achieved higher implementation rates (74% vs. 42%) compared to those in the standard class, with changes in attitudes, norms, and perceived control accounting for the variance in these outcomes	Enhance intentions and actual implementation of new behavior, resulting in significantly higher engagement
[28*] US	TPB	Quantitative, cross-sectional study	TPB was used to design educational interventions that targeted attitudes, perceived barriers, and knowledge to enhance intentions to adopt behavior	Intentions to adopt behavior were significantly influenced by perceived barriers and level of experience, with those having 6–10 years of experience being three times more likely to express intent, while the presence of perceived barriers nearly doubled the likelihood of diminished intent	Understanding the factors influencing intentions to adopt behavior, which facilitated the identification of specific barriers and predictors that could be addressed through targeted educational interventions
[35*] US	DoI	Quantitative, quasi-experimental pretest–posttest study with follow-up	DoI was used to develop two CE modules, participants completed a theory-based survey immediately before and after exposure to a randomly assigned module, and then again 3 weeks later	CE training, grounded in the DoI theory, successfully enhanced participants' knowledge and positive attitudes which in turn increased their intentions to change behavior	Understanding the behavior change process, guiding the development of the CE training content to address identified knowledge and practice gaps, by targeting specific constructs such as knowledge, attitudes, and behavioral intentions
[30*] Canada	DoI	Quantitative, cross-sectional study	DoI was used to developing a questionnaire	The survey findings reveal a number of opportunities for supporting adoption and effective use of CPD	Understand the various factors influencing the adoption of behavior, including identifying benefits and barriers associated with its implementation
[38*] Iran	TDF, TPB	Observational quantitative pre–post study	TDF was used to develop a questionnaire	Beliefs about consequences had the strongest influence on intention to adopt behavior, followed by beliefs about capabilities, highlighting the importance of addressing these psychological constructs	Effectively identifying key factors influencing behavior change, thereby validating its utility in understanding and promoting behavior adoption
[32*] Canada	TPB, TDF, & COM-B model	Non- randomized mixed methods	TPB was used for quantitative analysis, the TDF for qualitative and the COM-B model to triangulate findings analysis	The most frequent barriers to adopting behavior were related to skills and to social influence. Interestingly, the most frequent facilitators were also related to Skills	Influencing behavior by modifying intention and its psychosocial determinants. Innovation could change beliefs about capabilities by increasing knowledge about the desired behavior
[31*] Canada	TPB & TDF	Quantitative pre–post observational design	Both Godin’s Integrated Behavior Change Framework (an extension of the TPB) and the TDF were used to evaluate and understand both the quantitative (intention) and qualitative (behavior change) aspects of behavior following CPD courses	Moral norms, beliefs about capabilities, and beliefs about consequences significantly influenced intention to adopt behaviors post-CPD, supporting Godin’s Integrated Behavior Change Framework, while practical barriers such as lack of resources, highlighted by the TDF, contributed to the intention-behavior gap six months later	The use of theories provided a structured framework to evaluate the factors influencing behavior change, identify barriers and facilitators, and offer insights for designing more effective CPD interventions

## Discussion

### Summary of key findings

This scoping review aimed to explore the extent to which behavioral theories are utilized in the CPD of health professionals, focusing on understanding how these theories are applied and the impact they have on professional practice.

The review identified twelve studies that applied behavioral theories within the context of continuing professional development (CPD) for health professionals. The most frequently used were the Theory of Planned Behavior (TPB) and Self-Determination Theory (SDT), with TPB emerging as the predominant framework. These theories were mainly employed to examine health professionals’ attitudes, intentions, and behaviors related to CPD participation. Across studies, TPB was used to predict intentions to engage in CPD activities, whereas SDT emphasized the role of intrinsic motivation in sustaining ongoing learning. Despite their value, the integration of behavioral theory into CPD design and evaluation remains inconsistent. Many studies focused on using theory to explain participation and motivation rather than to inform the development, implementation, or evaluation of educational content. This suggests that, to date, behavioral theories have contributed more to understanding engagement in CPD than to shaping its pedagogical strategies or assessing learning outcomes.

### Strengths and limitations

This is the first scoping review to specifically examine the use of behavioral theories within CPD. A key strength of this review is its comprehensive approach, adhering to the PRISMA-ScR guidelines to ensure a thorough and systematic examination of the literature.

As Stewart and Klein [40] noted, the inconsistent reporting of theory across health research limits the comparability and utility of findings, a pattern echoed in this review as retrieved studies presented a challenge in synthesizing the findings. Inconsistencies mainly arose in three areas: (1) the absence of clear rationale for selecting a specific theory, (2) insufficient description of how theoretical constructs were operationalized within study design or data collection instruments, and (3) a lack of linkage between theoretical constructs and reported outcomes. Similar reporting gaps were identified by Hosseini et al. [41], who emphasized the lack of alignment between theory and intervention in CPD research. To mitigate against possible misinterpretations, reviewers frequently met to discuss study details that were lacking clarity, to reach consensus on their reporting in the context of the wider investigation.

Limitations include the exclusion of non-English studies, heterogeneity in theory application, and lack of standardized reporting across included studies. These factors constrained comparison and synthesis of findings.

### Interpretation of findings in relation to existing literature

This scoping review reveals a gap in the breadth and diversity of available research, which reflects findings from other healthcare domains, such as studies investigating adherence to clinical practice guidelines (CPGs) [42], adoption of antimicrobial stewardship initiatives [43] and medication error reporting [44]

Despite the importance of using behavioral theories in CPD, this review identified only 12 relevant studies, with a predominant focus on pharmacists (four studies), highlighting a considerable underrepresentation of other healthcare professionals.

The gap of inconsistent reporting of theory across the included studies Highlights the need for future studies to not only apply behavioral theories but also to clearly articulate how and why these frameworks are selected, ensuring that interventions are comprehensive and contextually relevant. These inconsistencies reflect challenges described more broadly in implementation research, where frameworks such as the Consolidated Framework for Implementation Research (CFIR) [45] have been proposed to establish a systematic linkage between context, mechanism, and outcome.

The current evidence base is limited by the predominance of cross-sectional and observational study designs, which restrict the ability to draw causal links between the use of behavioral theories and CPD outcomes. This is a well-documented challenge across CPD research more generally, as identified in recent scoping reviews [46, 47], which emphasize the need for more rigorous study designs, particularly randomized control trials (RCTs) and longitudinal studies, to better understand how CPD impacts both health professionals’ performance and patient outcomes.

The underutilization of more comprehensive frameworks like the TDF is a notable gap in the current literature, as this framework offers a broad range of behavioral determinants that can provide a more nuanced understanding of the multi-faceted factors influencing professional behavior in CPD contexts [48]. As Hakvoort et al. [49] highlighted in their scoping review on nursing CPD, the use of more holistic frameworks such as TDF can provide insights into how a wide range of factors,including social, environmental, and organizational affect professional development over time. To an extent, this has been demonstrated in a study by Allen et al. [50], who applied a social theory of learning to CPD programs, demonstrating how social, environmental, and organizational factors interact to shape learning and behavior change. Nevertheless, the potential advantage of these comprehensive frameworks remains theoretical until adequately tested in diverse CPD contexts.

This review also aligns with findings from Jeong et al. [7], who noted that CPD programs frequently struggle with inconsistent application of behavioral theories to guide both intervention design and data analysis. In many cases, theories are applied superficially to inform questionnaire design or outcome measurement but are not used to develop comprehensive, theory-based interventions. In addition, the increasing use of online and computer-based CPD programs, as highlighted by Hussein et al. [51], brings new challenges and opportunities. While technology-based education offers accessibility and flexibility, it requires careful integration of behavioral theories to ensure that learning is impactful and aligned with the professionals' practical needs. Jeong et al. [7] suggest that the effectiveness of online CPD can be enhanced by grounding these programs in solid theoretical frameworks, ensuring they address not only knowledge acquisition but also behavior change.

### Further work

Future research should prioritize more rigorous study designs, such as RCTs and longitudinal studies, to establish the causal impact of behavioral theories in CPD. Greater focus is needed on the theoretical justification of chosen frameworks, with an emphasis on multi-theoretical approaches like TDF. Additionally, studies should explore a wider range of healthcare professions beyond pharmacists, ensuring broader applicability. As technology-based CPD becomes more common, research should examine how behavioral theories can be integrated into online platforms to drive effective learning and behavior change. Finally, future work should focus on fully embedding behavioral theories into the design and implementation of CPD programs to promote sustained, theory-driven interventions.

## Conclusion

This review highlights critical gaps in literature regarding the use of behavioral theories in CPD. The limited number of studies, lack of methodological diversity, and insufficient theoretical justification suggest to a need for more rigorous, theoretically grounded research in this area. Future studies should aim to employ multi-theoretical frameworks such as the TDF to capture the complexities of CPD, ultimately leading to more effective, theory-informed interventions for health professionals' ongoing learning and development.

## Supplementary Information

Supplementary Material 1.

Supplementary Material 2.

## Data Availability

All data supporting the findings of this study are provided in the results section of the article. Additionally, supplementary files containing the complete search strategy and data extraction are available.
