# Supplementary material for: A scoping review of the use of behavioral theories in health professionals’ continuing professional development research
Source: BMC Med Educ. 2025 Dec 29;25:1721. doi: 10.1186/s12909-025-08276-3 (PMC12752388; doi:10.1186/s12909-025-08276-3)
Supplement: Supplementary file 2 — Supplementary Material 2. [file 12909_2025_8276_MOESM2_ESM.docx]

**PubMed:**

**29^th^ of August 2024**

**Category 1:**

| No. | Term | Results |
| --- | --- | --- |
| 1.1 | "veterinarian*"[Title/Abstract] | 17,000 |
| 1.2 | "social worker*"[Title/Abstract] | 13,900 |
| 1.3 | "physician*"[Title/Abstract] | 584,673 |
| 1.4 | "physiothera*"[Title/Abstract] | 38,975 |
| 1.5 | "pharmac*"[Title/Abstract] | 2,037,341 |
| 1.6 | "occupational therapist*"[Title/Abstract] | 8,680 |
| 1.7 | "nutritionist*"[Title/Abstract] | 5,199 |
| 1.8 | "nurse*"[Title/Abstract] | 440,883 |
| 1.9 | "mental health personnel*"[Title/Abstract] | 300 |
| 1.10 | "laboratory personnel*"[Title/Abstract] | 2,585 |
| 1.11 | "resident*"[Title/Abstract] | 355,584 |
| 1.12 | "health educator*"[Title/Abstract] | 3,765 |
| 1.13 | "midwive*"[Title/Abstract] | 20,000 |
| 1.14 | "doula*"[Title/Abstract] | 713 |
| 1.15 | "dentist*"[Title/Abstract] | 96,897 |
| 1.16 | "dietitian*"[Title/Abstract] | 9,754 |
| 1.17 | "anesthetist*"[Title/Abstract] | 5,908 |
| 1.18 | "alternative health practitioner*"[Title/Abstract] | 40 |
| 1.19 | "allied healthcare personnel*"[Title/Abstract] | 25 |
| 1.20 | "healthcare practitioner*"[Title/Abstract] | 4,270 |
| 1.21 | "healthcare profession*"[Title/Abstract] | 60,041 |
| 1.22 | "health personnel"[MeSH Terms] | 876,987 |
| 1.23 | "health personnel"[MeSH Terms] OR "health profession"[Title/Abstract] OR "health practitioner"[Title/Abstract] OR "allied healthcare personnel"[Title/Abstract] OR "alternative health practitioner"[Title/Abstract] OR "anesthetist"[Title/Abstract] OR "dietitian"[Title/Abstract] OR "dentist"[Title/Abstract] OR "doula"[Title/Abstract] OR "midwife"[Title/Abstract] OR "health educator"[Title/Abstract] OR "resident"[Title/Abstract] OR "laboratory personnel"[Title/Abstract] OR "mental health personnel"[Title/Abstract] OR "nurse"[Title/Abstract] OR "nutritionist"[Title/Abstract] OR "occupational therapist"[Title/Abstract] OR "pharmacist"[Title/Abstract] OR "physiotherapist"[Title/Abstract] OR "physician"[Title/Abstract] OR "social worker"[Title/Abstract] OR "veterinarian"[Title/Abstract] | 3,629,193 |

**Category 2:**

| No. | Term | Result |
| --- | --- | --- |
| 2.1 | "continued development"[Title] | 99 |
| 2.2 | "continued education"[Title] | 700 |
| 2.3 | "continued professional development"[Title] | 28 |
| 2.4 | "continued professional education"[Title] | 20 |
| 2.5 | "continuous education"[Title] | 206 |
| 2.6 | "continuous development"[Title] | 80 |
| 2.7 | "continuous professional education"[Title] | 89 |
| 2.8 | "continuous professional development"[Title] | 190 |
| 2.9 | "continuing professional education"[Title] | 300 |
| 2.10 | "continuing professional development"[Title] | 976 |
| 2.11 | "continuing development"[Title] | 104 |
| 2.12 | "continuing education"[Title] | 12,680 |
| 2.13 | "cpd"[Title] | 1280 |
| 2.14 | "professional development"[Title] | 5,666 |
| 2.15 | "professional development"[Title] OR "cpd"[Title] OR "continuing education"[Title] OR "continuing development"[Title] OR "continuing professional development"[Title] OR "continuing professional education"[Title] OR "continuous professional development"[Title] OR "continuous professional education"[Title] OR "continuous development"[Title] OR "continuous education"[Title] OR "continued professional education"[Title] OR "continued professional development"[Title] OR "continued education"[Title] OR "continued development"[Title] | 21,332 |

**Category 3:**

| No. | Term | Results |
| --- | --- | --- |
| 3.1 | "construct*"[Title/Abstract] | 570,314 |
| 3.2 | "concept*"[Title/Abstract] | 363,021 |
| 3.3 | "principle*"[Title/Abstract] | 310,136 |
| 3.4 | "framework*"[Title/Abstract] | 381,279 |
| 3.5 | "theor*"[Title/Abstract] | 485,737 |
| 3.6 | "theor"[Title/Abstract] OR "framework"[Title/Abstract] OR "principle"[Title/Abstract] OR "concept"[Title/Abstract] OR "construct"[Title/Abstract] | 3,447,510 |

**Category 4:**

| No. | Term | Results |
| --- | --- | --- |
| 4 | ("health personnel"[MeSH Terms] OR "healthcare profession*"[Title/Abstract] OR "healthcare practitioner*"[Title/Abstract] OR "allied healthcare personnel*"[Title/Abstract] OR "alternative health practitioner*"[Title/Abstract] OR "anesthetist*"[Title/Abstract] OR "dietitian*"[Title/Abstract] OR "dentist*"[Title/Abstract] OR "doula*"[Title/Abstract] OR "midwive*"[Title/Abstract] OR "health educator*"[Title/Abstract] OR "resident*"[Title/Abstract] OR "laboratory personnel*"[Title/Abstract] OR "mental health personnel*"[Title/Abstract] OR "nurse*"[Title/Abstract] OR "nutritionist*"[Title/Abstract] OR "occupational therapist*"[Title/Abstract] OR "pharmac*"[Title/Abstract] OR "physiothera*"[Title/Abstract] OR "physician*"[Title/Abstract] OR "social worker*"[Title/Abstract] OR "veterinarian*"[Title/Abstract]) AND ("professional development"[Title] OR "cpd"[Title] OR "continuing education"[Title] OR "continuing development"[Title] OR "continuing professional development"[Title] OR "continuing professional education"[Title] OR "continuous professional development"[Title] OR "continuous professional education"[Title] OR "continuous development"[Title] OR "continuous education"[Title] OR "continued professional education"[Title] OR "continued professional development"[Title] OR "continued education"[Title] OR "continued development"[Title]) AND ("theor*"[Title/Abstract] OR "framework*"[Title/Abstract] OR "principle*"[Title/Abstract] OR "concept*"[Title/Abstract] OR "construct*"[Title/Abstract]) | 722 |

**Embase:**

**29^th^ of August 2024**

**Category 1:**

| No. | Term | Results |
| --- | --- | --- |
| 1.1 | midwive*:ab,ti | 21,613 |
| 1.2 | veterinarian*:ab,ti | 17,484 |
| 1.3 | 'social worker*':ab,ti | 21,041 |
| 1.4 | physician*:ab,ti | 698,556 |
| 1.5 | physiothera*:ab,ti | 63,415 |
| 1.6 | pharmac*:ab,ti | 1,452,693 |
| 1.7 | 'occupational therapist*':ab,ti | 12,152 |
| 1.8 | nurse*:ab,ti | 418,133 |
| 1.9 | 'mental health  care personnel*':ab,ti | 9 |
| 1.10 | 'laboratory personnel*':ab,ti | 2,271 |
| 1.11 | resident*:ab,ti | 330,834 |
| 1.12 | 'health educator*':ab,ti | 3,093 |
| 1.13 | doula*:ab,ti | 628 |
| 1.14 | dentist*:ab,ti | 93,151 |
| 1.15 | dietitian*:ab,ti | 14,859 |
| 1.16 | anesthetist*:ab,ti | 6,752 |
| 1.17 | 'alternative health practitioner*':ab,ti | 41 |
| 1.18 | 'allied healthcare personnel*':ab,ti | 14 |
| 1.19 | 'healthcare practitioner*':ab,ti | 4,212 |
| 1.20 | 'healthcare profession*':ab,ti | 66,765 |
| 1.21 | 'health care personnel'/exp/mj | 668,450 |
| 1.22 | 'health personnel':ab,ti OR 'healthcare profession*':ab,ti OR 'healthcare practitioner*':ab,ti OR 'allied healthcare personnel*':ab,ti OR 'alternative health practitioner*':ab,ti OR anesthetist*:ab,ti OR dietitian*:ab,ti OR dentist*:ab,ti OR doula*:ab,ti OR 'health educator*':ab,ti OR resident*:ab,ti OR 'laboratory personnel*':ab,ti OR 'mental health  care personnel*':ab,ti OR nurse*:ab,ti OR 'occupational therapist*':ab,ti OR pharmac*:ab,ti OR physiothera*:ab,ti OR physician*:ab,ti OR 'social worker*':ab,ti OR veterinarian*:ab,ti OR midwive*:ab,ti OR 'health care personnel'/exp/mj | 3,359,082 |

**Category 2:**

| No. | Term | Results |
| --- | --- | --- |
| 2.1 | 'continued development':ti | 63 |
| 2.2 | 'continued education':ti | 346 |
| 2.3 | 'continued professional development':ti | 26 |
| 2.4 | 'continued professional education':ti | 12 |
| 2.5 | 'continuous education':ti | 139 |
| 2.6 | 'continuous development':ti | 59 |
| 2.7 | 'continuous professional education':ti | 13 |
| 2.8 | 'continuous professional development':ti | 146 |
| 2.9 | 'continuing professional education':ti | 171 |
| 2.10 | 'continuing professional development':ti | 1,250 |
| 2.11 | 'continuing development':ti | 65 |
| 2.12 | 'continuing education':ti | 7,690 |
| 2.13 | cpd:ti | 1,146 |
| 2.14 | 'professional development':ti | 3,730 |
| 2.15 | 'professional development':ti OR cpd:ti OR 'continuing education':ti OR 'continuing development':ti OR 'continuing professional development':ti OR 'continuing professional education':ti OR 'continuous professional development':ti OR 'continuous professional education':ti OR 'continuous development':ti OR 'continuous education':ti OR 'continued professional education':ti OR 'continued professional development':ti OR 'continued education':ti OR 'continued development':ti | 13,213 |

**Category 3:**

| No. | Term | Results |
| --- | --- | --- |
| 3.6 | construct*:ab,ti | 877,006 |
| 3.5 | concept*:ab,ti | 753,676 |
| 3.4 | principle*:ab,ti | 398,808 |
| 3.3 | framework*:ab,ti | 492,516 |
| 3.2 | theor*:ab,ti | 872,452 |
| 3.1 | theor*:ab,ti OR framework*:ab,ti OR principle*:ab,ti OR concept*:ab,ti OR construct*:ab,ti | 2,978,934 |

**Category 4:**

| No. | Term | Result |
| --- | --- | --- |
| 4 | ('professional development':ti OR cpd:ti OR 'continuing education':ti OR 'continuing development':ti OR 'continuing professional development':ti OR 'continuing professional education':ti OR 'continuous professional development':ti OR 'continuous professional education':ti OR 'continuous development':ti OR 'continuous education':ti OR 'continued professional education':ti OR 'continued professional development':ti OR 'continued education':ti OR 'continued development':ti) AND (theor*:ab,ti OR framework*:ab,ti OR principle*:ab,ti OR concept*:ab,ti OR construct*:ab,ti) AND ('health personnel':ab,ti OR 'healthcare profession*':ab,ti OR 'healthcare practitioner*':ab,ti OR 'allied healthcare personnel*':ab,ti OR 'alternative health practitioner*':ab,ti OR anesthetist*:ab,ti OR dietitian*:ab,ti OR dentist*:ab,ti OR doula*:ab,ti OR 'health educator*':ab,ti OR resident*:ab,ti OR 'laboratory personnel*':ab,ti OR 'mental health  care personnel*':ab,ti OR nurse*:ab,ti OR 'occupational therapist*':ab,ti OR pharmac*:ab,ti OR physiothera*:ab,ti OR physician*:ab,ti OR 'social worker*':ab,ti OR veterinarian*:ab,ti OR midwive*:ab,ti OR 'health care personnel'/exp/mj) | 755 |

**Cumulative Index of Nursing and Allied Health Literature (CINAHL):**

**28^th^ of August 2024**

**Category 1:**

| No. | Term | Results |
| --- | --- | --- |
| 1.1 | (MH "Health Personnel") | 57,131 |
| 1.2 | (MH "Allied Health Personnel") | 5,126 |
| 1.3 | TI health personnel* OR AB health personnel* | 5,319 |
| 1.4 | TI health profession* OR AB health profession* | 87,419 |
| 1.5 | TI health practitioner* OR AB health practitioner* | 13,627 |
| 1.6 | TI allied health personnel* OR AB allied health personnel* | 103 |
| 1.7 | TI alternative health practitioner* OR AB alternative health practitioner* | 79 |
| 1.8 | TI anesthetist* OR AB anesthetist* | 2,395 |
| 1.9 | TI dietitian* OR AB dietitian* | 6,100 |
| 1.10 | TI dentist* OR AB dentist* | 23,640 |
| 1.11 | TI doula* OR AB doula* | 840 |
| 1.12 | TI midwi* OR AB midwi* | 35,861 |
| 1.13 | TI health educator* OR AB health educator* | 4,563 |
| 1.14 | TI resident* OR AB resident* | 89,115 |
| 1.15 | TI laboratory personnel* OR AB laboratory personnel* | 496 |
| 1.16 | TI mental health personnel* OR AB mental health personnel* | 411 |
| 1.17 | TI nurse* OR AB nurse* | 386,769 |
| 1.18 | TI nutritionist* OR AB nutritionist* | 2,054 |
| 1.19 | TI occupational therapist* OR AB occupational therapist* | 12,356 |
| 1.20 | TI pharmac* OR AB pharmac* | 167,037 |
| 1.21 | TI physiothera* OR AB physiothera* | 27,420 |
| 1.22 | TI physician* OR AB physician* | 164,834 |
| 1.23 | TI social worker* OR AB social worker* | 16,690 |
| 1.24 | TI veterinarian* OR AB veterinarian* | 508 |
| 1.25 | S1 OR S2 OR S3 OR S4 OR S5 OR S6 OR S7 OR S8 OR S9 OR S10 OR S11 OR S12 OR S13 OR S14 OR S15 OR S16 OR S17 OR S18 OR S19 OR S20 OR S21 OR S22 OR S23 OR S24 | 973,810 |

**Category 2:**

| No. | Term | Results |
| --- | --- | --- |
| 2.1 | TI continu* professional development | 1,688 |
| 2.2 | TI cpd | 1,642 |
| 2.3 | TI professional development | 5,334 |
| 2.4 | TI continuing education | 9,838 |
| 2.5 | TI continuous education | 160 |
| 2.6 | TI continued education | 50 |
| 2.7 | TI continuing development | 1,683 |
| 2.8 | TI continuous development | 163 |
| 2.9 | TI continued development | 52 |
| 2.10 | TI continued professional education | 7 |
| 2.11 | TI continuing professional education | 434 |
| 2.12 | TI continuous professional education | 20 |
| 2.13 | S1 OR S2 OR S3 OR S4 OR S5 OR S6 OR S7 OR S8 OR S9 OR S10 OR S11 OR S12 | 16,946 |

**Category 3:**

| No. | Term | Results |
| --- | --- | --- |
| 3.1 | TI construct* OR AB construct* | 118,040 |
| 3.2 | TI concept* OR AB concept* | 166,336 |
| 3.3 | TI principle* OR AB principle* | 62,801 |
| 3.4 | TI framework* OR AB framework* | 118,561 |
| 3.5 | TI theor* OR AB theor* | 177,251 |
| 3.6 | S1 OR S2 OR S3 OR S4 OR S5 | 520,411 |

**Category 4:**

| No. | Term | Results |
| --- | --- | --- |
| 4 | 1.25 AND 2.13 AND 3.6 | 724 |

**Education Resources Information Center (ERIC):**

**28^th^ of August 2024**

**Category 1:**

| No. | Term | Results |
| --- | --- | --- |
| 1.1 | DE "Health Personnel" OR DE "Allied Health Personnel" OR DE "Mental Health Workers" OR DE "Nurses" OR DE "Physicians" OR DE "Psychologists" | 17,367 |
| 1.2 | TI health personnel* OR AB health personnel* | 3,620 |
| 1.3 | TI health profession* OR AB health profession* | 16,028 |
| 1.4 | TI health practitioner* OR AB health practitioner* | 3,191 |
| 1.5 | TI allied health personnel* OR AB allied health personnel* | 178 |
| 1.6 | TI alternative health practitioner* OR AB alternative health practitioner* | 91 |
| 1.7 | TI anesthetist* OR AB anesthetist* | 47 |
| 1.8 | TI dietitian* OR AB dietitian* | 134 |
| 1.9 | TI dentist* OR AB dentist* | 1,363 |
| 1.10 | TI doula* OR AB doula* | 16 |
| 1.11 | TI midwi* OR AB midwi* | 445 |
| 1.12 | TI health educator* OR AB health educator* | 6,492 |
| 1.13 | TI resident* OR AB resident* | 20,401 |
| 1.14 | TI laboratory personnel* OR AB laboratory personnel* | 796 |
| 1.15 | TI mental health personnel* OR AB mental health personnel* | 762 |
| 1.16 | TI nurse* OR AB nurse* | 10,692 |
| 1.17 | TI nutritionist* OR AB nutritionist* | 144 |
| 1.18 | TI occupational therapist* OR AB occupational therapist* | 826 |
| 1.19 | TI pharmac* OR AB pharmac* | 3,981 |
| 1.20 | TI physiothera* OR AB physiothera* | 315 |
| 1.21 | TI physician* OR AB physician* | 5,752 |
| 1.22 | TI social worker* OR AB social worker* | 8,768 |
| 1.23 | TI veterinarian* OR AB veterinarian* | 162 |
| 1.24 | S1 OR S2 OR S3 OR S4 OR S5 OR S6 OR S7 OR S8 OR S9 OR S10 OR S11 OR S12 OR S13 OR S14 OR S15 OR S16 OR S17 OR S18 OR S19 OR S20 OR S21 OR S22 OR S23 | 78,872 |

**Category 2:**

| No. | Term | Results |
| --- | --- | --- |
| 2.1 | TI continu* professional development | 534 |
| 2.2 | TI cpd | 99 |
| 2.3 | TI professional development | 11,952 |
| 2.4 | TI continuing education | 3,795 |
| 2.5 | TI continuous education | 173 |
| 2.6 | TI continued education | 68 |
| 2.7 | TI continuing development | 588 |
| 2.8 | TI continuous development | 140 |
| 2.9 | TI continued development | 40 |
| 2.10 | TI continued professional education | 4 |
| 2.11 | TI continuing professional education | 463 |
| 2.12 | TI continuous professional education | 13 |
| 2.13 | S1 OR S2 OR S3 OR S4 OR S5 OR S6 OR S7 OR S8 OR S9 OR S10 OR S11 OR S12 | 16,047 |

**Category 3:**

| No. | Term | Results |
| --- | --- | --- |
| 3.1 | TI construct* OR AB construct* | 105,969 |
| 3.2 | TI concept* OR AB concept* | 197,927 |
| 3.3 | TI principle* OR AB principle* | 58,781 |
| 3.4 | TI framework* OR AB framework* | 104,642 |
| 3.5 | TI theor* OR AB theor* | 194,189 |
| 3.6 | S1 OR S2 OR S3 OR S4 OR S5 | 502,458 |

**Category 4:**

| No. | Term | Results |
| --- | --- | --- |
| 4 | 1.24 AND 2.13 AND 3.6 | 345 |

**APA PsycArticles:**

**28^th^ of August 2024**

**Category 1:**

| No. | Term | Results |
| --- | --- | --- |
| 1.1 | MAINSUBJECT.EXACT("Health Personnel") AND PEER(yes) | 2,471 |
| 1.2 | MAINSUBJECT.EXACT("Allied Health Personnel") AND PEER(yes) | 81 |
| 1.3 | noft(health personnel*) AND PEER(yes) | 15,394 |
| 1.4 | noft(health* profession*) AND PEER(yes) | 16,675 |
| 1.5 | noft(health* practitioner*) AND PEER(yes) | 2,366 |
| 1.6 | noft(allied health personnel*) AND PEER(yes) | 219 |
| 1.7 | noft(alternative health practitioner*) AND PEER(yes) | 86 |
| 1.8 | noft(anesthetist*) AND PEER(yes) | 4 |
| 1.9 | noft(dietitian*) AND PEER(yes) | 7 |
| 1.10 | noft(dentist*) AND PEER(yes) | 532 |
| 1.11 | noft(doula*) AND PEER(yes) | 2 |
| 1.12 | noft(midwi*) AND PEER(yes) | 124 |
| 1.13 | noft(health educator*) AND PEER(yes) | 496 |
| 1.14 | noft(resident*) AND PEER(yes) | 2,252 |
| 1.15 | noft(laboratory personnel*) AND PEER(yes) | 505 |
| 1.16 | noft(mental health personnel*) AND PEER(yes) | 13,231 |
| 1.17 | noft(nurse*) AND PEER(yes) | 1,215 |
| 1.18 | noft(nutritionist*) AND PEER(yes) | 5 |
| 1.19 | noft(occupational therapist*) AND PEER(yes) | 142 |
| 1.20 | noft(pharmac*) AND PEER(yes) | 743 |
| 1.21 | noft(physiothera*) AND PEER(yes) | 54 |
| 1.22 | noft(physician*) AND PEER(yes) | 2,339 |
| 1.23 | noft(social worker*) AND PEER(yes) | 2,125 |
| 1.24 | noft(veterinarian*) AND PEER(yes) | 13 |
| 1.25 | (noft(veterinarian*) AND PEER(yes)) OR (noft(social worker*) AND PEER(yes)) OR (noft(physician*) AND PEER(yes)) OR (noft(physiothera*) AND PEER(yes)) OR (noft(pharmac*) AND PEER(yes)) OR (noft(occupational therapist*) AND PEER(yes)) OR (noft(nutritionist*) AND PEER(yes)) OR (noft(nurse*) AND PEER(yes)) OR (noft(mental health personnel*) AND PEER(yes)) OR (noft(laboratory personnel*) AND PEER(yes)) OR (noft(resident*) AND PEER(yes)) OR (noft(health educator*) AND PEER(yes)) OR (noft(doula*) AND PEER(yes)) OR (noft(dentist*) AND PEER(yes)) OR (noft(dietitian*) AND PEER(yes)) OR (noft(anesthetist*) AND PEER(yes)) OR (noft(alternative health practitioner*) AND PEER(yes)) OR (noft(allied health personnel*) AND PEER(yes)) OR (noft(health* practitioner*) AND PEER(yes)) OR (noft(health* profession*) AND PEER(yes)) OR (noft(health personnel*) AND PEER(yes)) OR (MAINSUBJECT.EXACT("Allied Health Personnel") AND PEER(yes)) OR (MAINSUBJECT.EXACT("Health Personnel") AND PEER(yes)) OR (noft(midwi*) AND PEER(yes)) | 30,758 |

**Category 2:**

| No. | Term | Results |
| --- | --- | --- |
| 2.1 | title(continu* professional development) AND PEER(yes) | 6 |
| 2.2 | title(cpd) AND PEER(yes) | 1 |
| 2.3 | title(professional development) AND PEER(yes) | 87 |
| 2.4 | title(continuing education) AND PEER(yes) | 65 |
| 2.5 | title(continuous education) AND PEER(yes) | 1 |
| 2.6 | title(continued education) AND PEER(yes) | 1 |
| 2.7 | title(continuing development) AND PEER(yes) | 7 |
| 2.8 | title(continuous development) AND PEER(yes) | 1 |
| 2.9 | title(continued development) AND PEER(yes) | 2 |
| 2.10 | title(continued professional education) AND PEER(yes) | 0 |
| 2.11 | title(continuing professional education) AND PEER(yes) | 9 |
| 2.12 | title(continuous professional education) AND PEER(yes) | 0 |
| 2.13 | (title(continu* professional development) AND PEER(yes)) OR (title(cpd) AND PEER(yes)) OR (title(professional development) AND PEER(yes)) OR (title(continuing education) AND PEER(yes)) OR (title(continuous education) AND PEER(yes)) OR (title(continued education) AND PEER(yes)) OR (title(continuing development) AND PEER(yes)) OR (title(continuous development) AND PEER(yes)) OR (title(continued development) AND PEER(yes)) OR (title(continued professional education) AND PEER(yes)) OR (title(continuing professional education) AND PEER(yes)) OR (title(continuous professional education) AND PEER(yes)) | 158 |

**Category 3:**

| No. | Term | Results |
| --- | --- | --- |
| 3.1 | noft(construct*) AND PEER(yes) | 20,610 |
| 3.2 | noft(concept*) AND PEER(yes) | 23,996 |
| 3.3 | noft(principle*) AND PEER(yes) | 6,836 |
| 3.4 | noft(framework*) AND PEER(yes) | 8,131 |
| 3.5 | noft(theor*) AND PEER(yes) | 42,964 |
| 3.6 | (noft(construct*) AND PEER(yes)) OR (noft(concept*) AND PEER(yes)) OR (noft(principle*) AND PEER(yes)) OR (noft(framework*) AND PEER(yes)) OR (noft(theor*) AND PEER(yes)) | 80,628 |

**Category 4:**

| No. | Term | Results |
| --- | --- | --- |
| 4 | ((noft(veterinarian*) AND PEER(yes)) OR (noft(social worker*) AND PEER(yes)) OR (noft(physician*) AND PEER(yes)) OR (noft(physiothera*) AND PEER(yes)) OR (noft(pharmac*) AND PEER(yes)) OR (noft(occupational therapist*) AND PEER(yes)) OR (noft(nutritionist*) AND PEER(yes)) OR (noft(nurse*) AND PEER(yes)) OR (noft(mental health personnel*) AND PEER(yes)) OR (noft(laboratory personnel*) AND PEER(yes)) OR (noft(resident*) AND PEER(yes)) OR (noft(health educator*) AND PEER(yes)) OR (noft(doula*) AND PEER(yes)) OR (noft(dentist*) AND PEER(yes)) OR (noft(dietitian*) AND PEER(yes)) OR (noft(anesthetist*) AND PEER(yes)) OR (noft(alternative health practitioner*) AND PEER(yes)) OR (noft(allied health personnel*) AND PEER(yes)) OR (noft(health* practitioner*) AND PEER(yes)) OR (noft(health* profession*) AND PEER(yes)) OR (noft(health personnel*) AND PEER(yes)) OR (MAINSUBJECT.EXACT("Allied Health Personnel") AND PEER(yes)) OR (MAINSUBJECT.EXACT("Health Personnel") AND PEER(yes)) OR (noft(midwi*) AND PEER(yes))) AND ((title(continu* professional development) AND PEER(yes)) OR (title(cpd) AND PEER(yes)) OR (title(professional development) AND PEER(yes)) OR (title(continuing education) AND PEER(yes)) OR (title(continuous education) AND PEER(yes)) OR (title(continued education) AND PEER(yes)) OR (title(continuing development) AND PEER(yes)) OR (title(continuous development) AND PEER(yes)) OR (title(continued development) AND PEER(yes)) OR (title(continued professional education) AND PEER(yes)) OR (title(continuing professional education) AND PEER(yes)) OR (title(continuous professional education) AND PEER(yes))) AND ((noft(construct*) AND PEER(yes)) OR (noft(concept*) AND PEER(yes)) OR (noft(principle*) AND PEER(yes)) OR (noft(framework*) AND PEER(yes)) OR (noft(theor*) AND PEER(yes))) | 25 |

**ProQuest Database**

**29^th^ of August 2024**

**Category 1:**

| No. | Term | Results |
| --- | --- | --- |
| 1.0 | MESH.EXACT("Health Personnel") AND PEER(yes) | 17,904 |
| 1.1 | MESH.EXACT("Allied Health Personnel") AND PEER(yes) | 754 |
| 1.2 | noft(health personnel*) AND PEER(yes) | 195,103 |
| 1.3 | noft(health profession*) AND PEER(yes) | 383,912 |
| 1.4 | noft(health practitioner*) AND PEER(yes) | 126,226 |
| 1.5 | noft(allied health personnel*) AND PEER(yes) | 8,005 |
| 1.6 | noft(Alternative Health Practitioner*) AND PEER(yes) | 5,329 |
| 1.7 | noft(anesthetist*) AND PEER(yes) | 5,898 |
| 1.8 | noft(dietitian*) AND PEER(yes) | 11,182 |
| 1.9 | noft(dentist*) AND PEER(yes) | 157,527 |
| 1.10 | noft(doula*) AND PEER(yes) | 1,449 |
| 1.11 | noft(midwi*) AND PEER(yes) | 57,768 |
| 1.12 | noft(health educator*) AND PEER(yes) | 33,036 |
| 1.13 | noft(resident*) AND PEER(yes) | 322,510 |
| 1.14 | noft(laboratory personnel*) AND PEER(yes) | 14,799 |
| 1.15 | noft(mental health personnel*) AND PEER(yes) | 46,660 |
| 1.16 | noft(nurse*) AND PEER(yes) | 397,247 |
| 1.17 | noft(nutritionist*) AND PEER(yes) | 4,605 |
| 1.18 | noft(occupational therapist*) AND PEER(yes) | 14,099 |
| 1.19 | noft(pharmac*) AND PEER(yes) | 629,678 |
| 1.20 | noft(physiothera*) AND PEER(yes) | 46,116 |
| 1.21 | noft(physician*) AND PEER(yes) | 460,660 |
| 1.22 | noft(social worker*) AND PEER(yes) | 225,259 |
| 1.23 | noft(veterinarian*) AND PEER(yes) | 12,577 |
| 1.24 | (MESH.EXACT("Health Personnel") AND PEER(yes)) OR (MESH.EXACT("Allied Health Personnel") AND PEER(yes)) OR (noft(health personnel*) AND PEER(yes)) OR (noft(health profession*) AND PEER(yes)) OR (noft(health practitioner*) AND PEER(yes)) OR (noft(allied health personnel*) AND PEER(yes)) OR (noft(Alternative Health Practitioner*) AND PEER(yes)) OR (noft(anesthetist*) AND PEER(yes)) OR (noft(dietitian*) AND PEER(yes)) OR (noft(dentist*) AND PEER(yes)) OR (noft(doula*) AND PEER(yes)) OR (noft(midwi*) AND PEER(yes)) OR (noft(health educator*) AND PEER(yes)) OR (noft(resident*) AND PEER(yes)) OR (noft(laboratory personnel*) AND PEER(yes)) OR (noft(mental health personnel*) AND PEER(yes)) OR (noft(nurse*) AND PEER(yes)) OR (noft(nutritionist*) AND PEER(yes)) OR (noft(occupational therapist*) AND PEER(yes)) OR (noft(pharmac*) AND PEER(yes)) OR (noft(physiothera*) AND PEER(yes)) OR (noft(physician*) AND PEER(yes)) OR (noft(social worker*) AND PEER(yes)) OR (noft(veterinarian*) AND PEER(yes)) | 2,521,861 |

**Category 2:**

| No. | Term | Results |
| --- | --- | --- |
| 2.1 | title(continu* professional development) AND PEER(yes) | 1,984 |
| 2.2 | title(cpd) AND PEER(yes) | 1,235 |
| 2.3 | title(professional development) AND PEER(yes) | 19,586 |
| 2.4 | title(continuing education) AND PEER(yes) | 8,087 |
| 2.5 | title(continuous education) AND PEER(yes) | 387 |
| 2.6 | title(continued education) AND PEER(yes) | 152 |
| 2.7 | title(continuing development) AND PEER(yes) | 2,096 |
| 2.8 | title(continuous development) AND PEER(yes) | 1,004 |
| 2.9 | title(continued development) AND PEER(yes) | 175 |
| 2.10 | title(continued professional education) AND PEER(yes) | 11 |
| 2.11 | title(continuing professional education) AND PEER(yes) | 846 |
| 2.12 | title(continuous professional education) AND PEER(yes) | 34 |
| 2.13 | ((title(continu* professional development) AND PEER(yes)) OR (title(cpd) AND PEER(yes)) OR (title(professional development) AND PEER(yes)) OR (title(continuing education) AND PEER(yes)) OR (title(continuous education) AND PEER(yes)) OR (title(continued education) AND PEER(yes)) OR (title(continuing development) AND PEER(yes)) OR (title(continuous development) AND PEER(yes)) OR ((title(continued development) AND PEER(yes)) AND RO AND (title(continued professional education) AND PEER(yes)))) OR (title(continuing professional education) AND PEER(yes)) OR (title(continuous professional education) AND PEER(yes)) | 30,140 |

**Category 3:**

| No. | Term | Results |
| --- | --- | --- |
| 3.1 | noft(construct*) AND PEER(yes) | 1,365,553 |
| 3.2 | noft(concept*) AND PEER(yes) | 1,410,343 |
| 3.3 | noft(principle*) AND PEER(yes) | 567,916 |
| 3.4 | noft(framework*) AND PEER(yes) | 1,053,162 |
| 3.5 | noft(theor*) AND PEER(yes) | 2,619,264 |
| 3.6 | (noft(construct*) AND PEER(yes)) OR (noft(concept*) AND PEER(yes)) OR (noft(principle*) AND PEER(yes)) OR (noft(framework*) AND PEER(yes)) OR (noft(theor*) AND PEER(yes)) | 5,744,824 |

**Category 4:**

| No. | Term | Results |
| --- | --- | --- |
| 4 | ((MESH.EXACT("Health Personnel") AND PEER(yes)) OR (MESH.EXACT("Allied Health Personnel") AND PEER(yes)) OR (noft(health personnel*) AND PEER(yes)) OR (noft(health profession*) AND PEER(yes)) OR (noft(health practitioner*) AND PEER(yes)) OR (noft(allied health personnel*) AND PEER(yes)) OR (noft(Alternative Health Practitioner*) AND PEER(yes)) OR (noft(anesthetist*) AND PEER(yes)) OR (noft(dietitian*) AND PEER(yes)) OR (noft(dentist*) AND PEER(yes)) OR (noft(doula*) AND PEER(yes)) OR (noft(midwi*) AND PEER(yes)) OR (noft(health educator*) AND PEER(yes)) OR (noft(resident*) AND PEER(yes)) OR (noft(laboratory personnel*) AND PEER(yes)) OR (noft(mental health personnel*) AND PEER(yes)) OR (noft(nurse*) AND PEER(yes)) OR (noft(nutritionist*) AND PEER(yes)) OR (noft(occupational therapist*) AND PEER(yes)) OR (noft(pharmac*) AND PEER(yes)) OR (noft(physiothera*) AND PEER(yes)) OR (noft(physician*) AND PEER(yes)) OR (noft(social worker*) AND PEER(yes)) OR (noft(veterinarian*) AND PEER(yes))) AND (((title(continu* professional development) AND PEER(yes)) OR (title(cpd) AND PEER(yes)) OR (title(professional development) AND PEER(yes)) OR (title(continuing education) AND PEER(yes)) OR (title(continuous education) AND PEER(yes)) OR (title(continued education) AND PEER(yes)) OR (title(continuing development) AND PEER(yes)) OR (title(continuous development) AND PEER(yes)) OR ((title(continued development) AND PEER(yes)) AND RO AND (title(continued professional education) AND PEER(yes)))) OR (title(continuing professional education) AND PEER(yes)) OR (title(continuous professional education) AND PEER(yes))) AND ((noft(construct*) AND PEER(yes)) OR (noft(concept*) AND PEER(yes)) OR (noft(principle*) AND PEER(yes)) OR (noft(framework*) AND PEER(yes)) OR (noft(theor*) AND PEER(yes))) AND PEER(yes) | 1,480 |
